# Electroacupuncture posttreatment attenuates the inflammatory injury in rats after MIRI through PPARγ/NF-κb pathway

**DOI:** 10.3389/fcvm.2025.1562285

**Published:** 2025-04-23

**Authors:** Xinxue Jiang, Chuchu Shen, Xing Li, Xuefeng Xia, Senlei Xu, Hongru Zhang

**Affiliations:** ^1^ College of Acupuncture and Tuina-Nurturing and Rehabilitation, Nanjing University of Traditional Chinese Medicine, Nanjing, Jiangsu, China; ^2^ Laboratory of Acupuncture and Medicine Combination, Nanjing University of Chinese Medicine, Nanjing, Jiangsu, China; ^3^ Tanan Shuyuan, Nanjing University of Traditional Chinese Medicine, Nanjing, Jiangsu, China

**Keywords:** myocardial ischemia-reperfusion injury, electroacupuncture, inflammation, macrophage M2 polarization, PPARγ/NF-κB pathway

## Abstract

**Objective:**

This study aims to investigate the activation of the PPARγ/NF-κB pathway and its influence on macrophage M2 polarization induced by acupuncture at the Neiguan acupoint. Additionally, it seeks to explore the potential mechanisms by which electroacupuncture treatment may facilitate the reduction of inflammation in rats subjected to myocardial ischemia-reperfusion injury (MIRI).

**Methods:**

SD rats were randomly assigned to four groups: sham operation, model, electroacupuncture, and inhibitor, with 12 rats in each group. The left anterior descending branch of the coronary artery was ligated to establish the MIRI rat model. Electroacupuncture intervention at the bilateral Neiguan acupoints commenced 24 h post-chest closure, lasting for 30 min once daily over three consecutive days. Myocardial infarction was assessed through electrocardiography, while cardiac function was evaluated via echocardiography 24 h after modeling. The morphology and structure of myocardial tissues were examined using HE staining. Myocardial tissue levels of interleukin-6 (IL-6), tumor necrosis factor-α (TNF-α), interleukin-10 (IL-10) and transforming growth factor-β (TGF-β) were measured by ELISA in each group of rats. The expression of the M1 macrophage marker inducible nitric oxide synthase (iNOS) and the M2 macrophage marker arginase-1 (Arg-1) in myocardial tissues was analyzed using immunohistochemistry (IHC). Detection of macrophage polarization status in myocardial tissue by flow cytometry. Additionally, peroxisome proliferator-activated receptor gamma (PPARγ) and nuclear transcription factor-κB (NF-κB) expression in myocardial tissues was assessed using Western blotting (WB).

**Results:**

Compared with the sham-operated group, rats in the model group exhibited a significant decrease in ejection fraction (EF) (*P* < 0.01), along with notable myocardial fiber damage characterized by inflammatory cell infiltration. Additionally, there was an elevated expression of IL-6, TGF-β, iNOS, CD-86 and phosphorylated p65 (p-p65) in myocardial tissue (*P* < 0.01, *P* < 0.01, *P* < 0.05, *P* < 0.05 and *P* < 0.01, respectively). In contrast, rats in the electroacupuncture group demonstrated an increase in EF (*P* < 0.01) compared to the model group. Myocardial fiber damage was significantly ameliorated, inflammatory cell infiltration was reduced. Furthermore, the expression of IL-10, TGF-β, Arg-1, CD-163 and PPARγ in cardiac muscle tissue was increased (*P* < 0.01, *P* < 0.01, *P* < 0.05, *P* < 0.05 and *P* < 0.01, respectively). Conversely, when compared to the electroacupuncture group, the EF of rats in the inhibitor group was significantly reduced (*P* < 0.05), with pronounced myocardial fiber damage and accompanying inflammatory cell infiltration. Additionally, IL-6, TGF-β, iNOS CD-86 and p-p65 expression in myocardial tissue was increased (*P* < 0.01, *P* < 0.01, *P* < 0.05, *P* < 0.05 and *P* < 0.01, respectively).

**Conclusion:**

Electroacupuncture may ameliorate myocardial MIRI by activating the PPARγ/NF-κB pathway, promoting polarization of macrophages towards the M2 type, and reducing inflammatory damage in myocardial tissues.

## 1 Introduction

Ischemic heart disease (IHD) ranks among the leading causes of death and disability globally (1–3). Early reperfusion is critical for enhancing the survival of ischemic cardiomyocytes; however, reperfusion can also accelerate and worsen myocardial injury, a phenomenon known as myocardial ischemia/reperfusion injury (MIRI) (4–6). The initial inflammatory environment instigates angiogenesis, with macrophages playing a crucial and sustained role in the vascular pumping and remodeling processes. Macrophages are generally categorized based on their activation status into M1 macrophages, which exhibit pro-inflammatory characteristics (F4/80+CD86+), and M2 macrophages, which possess anti-inflammatory properties (F4/80+CD163+) (7, 8). Research indicates that distinct macrophage phenotypes drive angiogenesis (9). Additionally, PPARγ exerts a negative regulatory effect on both immune and inflammatory responses. It can bind to NF-κB, preventing its nuclear translocation, thereby antagonizing the NF-κB signaling pathway, reducing the inflammatory response, and providing protective effects for the organism. The PPARγ and NF-κB signaling pathways play crucial roles in macrophage polarization, with their interactions regulating the transition of macrophages from a pro-inflammatory (M1-type) to an anti-inflammatory (M2-type) phenotype. PPARγ exerts an anti-inflammatory effect by inhibiting the NF-κB signaling pathway, which promotes M2-type polarization, while the activation of NF-κB favors M1-type polarization (10). Activation of the PPARγ/NF-κB signaling pathway enhances the expression of aortic PPARγ protein while decreasing NF-κB protein levels. This modulation leads to a notable reduction in the M1-type phenotypic marker iNOS and a significant increase in the M2-type phenotypic marker Arg-1, indicating that the PPARγ/NF-κB signaling pathway facilitates M2-type macrophage polarization by inhibiting the polarization of M1-type macrophages, ultimately contributing to the mitigation of MIRI (11).

Acupuncture and moxibustion represent a significant component of traditional Chinese medicine, demonstrating unique efficacy in the treatment of coronary heart disease and other cardiovascular conditions. The theoretical foundations of these practices can be traced back to the “Yellow Emperor's Classic of Internal Medicine,” which established the theory of meridian identification. This theory posits that acupuncture can achieve therapeutic outcomes by clearing the meridians and regulating qi and blood through adjustments to the human body's meridian system. Recent studies have elucidated the mechanisms by which acupuncture exerts its therapeutic effects in the treatment of myocardial ischemia-reperfusion injury (MIRI). Evidence suggests that acupuncture can significantly mitigate myocardial injury and enhance cardioprotection through the modulation of various signaling pathways. These findings not only establish a scientific foundation for the use of acupuncture in MIRI management but also indicate avenues for the further optimization of treatment protocols. Experimental studies have confirmed that electroacupuncture preconditioning significantly down-regulates reactive oxygen species (ROS) levels in rat myocardial tissues within the myocardial ischemia-reperfusion injury (MIRI) model, while also enhancing cardiac function indices (12). Electroacupuncture therapy has shown significant therapeutic potential in modulating the inflammatory response and providing myocardial protection following myocardial infarction, owing to its multi-target and multi-pathway action characteristics (13). Han Yongli et al. investigated the impact of acupoint pretreatment on calcium homeostasis as observed through MRI in diabetic rats, focusing on the influence of specimen pretreatment on MRI outcomes in this population (14). Experimental data indicate that stimulating the Neiguan acupoint effectively regulates cellular autophagy and inhibits programmed cell death, thereby alleviating ischemic lesions in myocardial tissues (15, 16). The results indicate that electroacupuncture pretreatment at the Neiguan acupoints effectively inhibits the mitochondrial autophagy process by modulating the mTORC1-ULK1-FUNDC1 signaling pathway. This modulation subsequently enhances mitochondrial energy supply and improves energy metabolism following myocardial ischemia-reperfusion injury (17).

As a significant point on the hand's syncope Yin pericardium meridian, Neiguan's unique anatomical location and its connections within the meridian system render it crucial in clinical Chinese medicine. This point is situated on the palmar side of the forearm, approximately 2 inches above the transverse wrist line. It belongs to the Pericardium meridian associated with the hand's syncope Yin and is also linked to the Sanjiao meridian of the hand's Shaoyang, as well as the Yinwei vessel. From a nomenclature perspective, the term “Neiguan” signifies its clinical role in “opening the chest and diaphragm, alleviating obstructions.” As stated in ancient texts, “the heart and chest Neiguan seeks,” which underscores its unique significance in the treatment of cardiovascular diseases (18, 19). The analysis of meridian theory reveals a significant similarity between the pericardium meridian associated with hand syncope and the cardiac meridian of the lesser yin of the hand, particularly in their circulation routes and the distribution of acupoints. Moreover, the anatomical characteristics of the pericardium, which envelops the heart, suggest a physiological and pathological interdependence between the two. This relationship provides a theoretical foundation for utilizing the Neiguan acupoints in the treatment of cardiac diseases. The primary therapeutic function of this acupuncture point has been extensively documented by medical practitioners throughout history. Classic texts such as “Preparing for the Emergency—Thousand Golden Essentials” and “Acupuncture and Moxibustion Dacheng” have emphasized its effectiveness in treating conditions such as heart-related pain, heart deficiency, and headaches. These works highlight the point's unique advantages in addressing cardiopulmonary deficiencies and solid diseases (20). Due to these properties, the Neiguan point is a crucial aspect of clinical treatment for cardiac diseases, playing an indispensable role in the treatment of cardiac system disorders.

The present experiment aimed to investigate the effects of electroacupuncture at the Neiguan point on myocardial ischemia-reperfusion injury (MIRI) in rats. This study focuses on the activation of the PPARγ/NF-κB signaling pathway to modulate macrophage M2-type polarization, thereby mitigating myocardial inflammatory injury.

## 2 Materials and methods

### 2.1 Experimental animals and grouping

Forty-eight SPF-grade male SD rats were obtained from Beijing Viton Lihua Laboratory Animal Technology Co. at the age of 7 weeks, with a mass ranging from 240 to 280 grams. The rats were housed for one week in the SPF-grade laboratory animal center of Nanjing University of Traditional Chinese Medicine, where they were maintained on a 12-h light/12-h darkness cycle, with free access to food. The environmental conditions were controlled at a room temperature of (25 ± 2) °C and a humidity of (50 ± 5)%. Following this adaptive rearing period, rats with relatively high variability were excluded from the study using electrocardiogram and echocardiogram assessments. The remaining rats were randomly assigned to four groups: sham operation group, model group, model + electroacupuncture group, and model + electroacupuncture + GW9962 group, utilizing a random number table method for allocation. The treatment of animals throughout the experiment adhered to the “Guiding Opinions on the Kind Treatment of Laboratory Animals.”

### 2.2 Main instruments and reagents

Small animal ventilator, isoflurane anesthesia machine (Shenzhen Reward Life Science and Technology Co., Ltd.), cardiac monitor (Power-lab, Australia), Mylab Doppler ultrasound (ESAOTE, Italy), microscope (Olympus, Japan), desktop high-speed freezing centrifuge (Shanghai Anting Scientific Instrument Factory), pathological section machine (Leica, Germany), enzyme labeling instrument (Biotek, USA), electrophoresis instrument, electrophoresis tank (Bio-rad, USA), gel imaging system (Protein Simple, USA). Biotek (USA), electrophoresis instrument and electrophoresis tank (Bio-rad, USA), gel imaging system (Protein Simple, USA), Electronic balance (METTER AE100, Switzerland), Han's electroacupuncture instrument (Nanjing Jisheng Medical Technology Co., Ltd.), Huatuo brand disposable stainless steel acupuncture needles (Suzhou Medical Supplies Factory Co., Ltd.). Isoflurane (Shenzhen Reward Life Science and Technology Co., Ltd.), RIPA lysate (Shanghai Biyuntian Biotechnology Co., Ltd.), Protein Maker (W20350ES72, Shanghai Yi Sheng Bio-technology Co., Ltd.), ECL luminescent liquid (Shanghai Tianneng Technology Co., Ltd.), BCA protein quantification kit (Thermo, USA), primary antibodies PPARγ, p-p65, iNOS and Arg-1 (Wuhan Three Eagles Biotechnology Co., Ltd.) HRP-labeled goat anti-rabbit IgG secondary antibody and HRP-labeled goat anti-mouse IgG secondary antibody (SA00001-1, SA00001-2, Wuhan Three Eagles Biotechnology Co., Ltd.), IL-6ELISA kit (Catalog No.: AF20240407), etc.

### 2.3 Molding method

According to the method of establishing myocardial ischemia-reperfusion model in the preliminary stage of our group, rats were anesthetized using 5% isoflurane mixed with 70% nitrogen and 30% oxygen, maintaining a concentration of 1%-2%. The rats were positioned supine on an experimental plate maintained at (37 ± 3) °C. Tracheal intubation and mechanical ventilation were performed using a ventilator set to a respiratory rate of 65–70 breaths per min and a tidal volume of 1.0 ml per 100 mg of body weight. A cardiac monitor was connected, and the electrocardiogram was monitored using a standard II lead. The chest cavity was opened along the fourth and fifth intercostal spaces, and the left anterior descending branch (LAD) of the coronary artery was ligated with a 6.0 silk ligature, approximately 2 mm in width and depth. After 30 min of ischemia, the silk ligature was released to allow for reperfusion. The chest was then closed and sutured layer by layer, after which the ventilator was disconnected and respiratory secretions were cleared. In the electroacupuncture + GW9962 group, rats received an intraperitoneal injection of 10 mg/kg of GW9962 solution 1 h prior to modeling. In the sham-operated group, only the wire was threaded without ligation.

**Figure 1:**
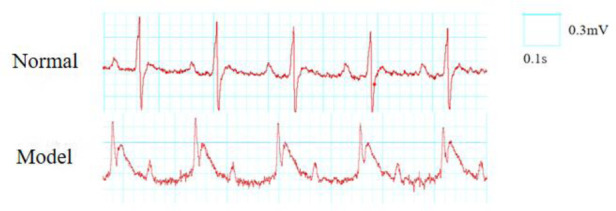
ECG changes before and after MIRI in rats (lead II).

Criteria for successful modeling: myocardial ischemia is indicated by bow-back elevation of the ST segment in lead II of the limb during ischemia, elevation of the T wave, and paleness or cyanosis on the surface of the heart; reperfusion is considered successful if the ST segment falls back by more than 1/2 after reperfusion, and if the surface of the heart turns red in the area of pallor (17). Refer to Figure 1.

### 2.4 Intervention methodology

The electroacupuncture procedure was conducted as follows: both the electroacupuncture group and the electroacupuncture + GW9962 group began treatment 24 h after chest closure, continuing for three consecutive days. Each session lasted 30 min and utilized 0.18 mm × 13 mm disposable stainless steel needles designed for bipolar electroacupuncture. Needles were inserted straight into the bilateral Neiguan point (PC6) to a depth of 2–3 mm and connected to Han's Electroacupuncture Instrument, operating at frequencies of 2 Hz and 100 Hz, with a current intensity set at 2 mA. The method for acupoint localization was based on guidelines from “*Experimental Acupuncture and Moxibustion*” (Yu Shuguang, Xu Bin, Beijing: People's Health Press, 2012). The sham operation group and the model group were maintained under standard conditions and subjected to the same grasping and fixation techniques.

### 2.5 Observation indicators and testing methods

Echocardiographic assessment of cardiac function changes in rats was performed using Doppler ultrasonography, with data collected 24 h post-modeling for each group. The rats were anesthetized with 5% isoflurane, maintained at a concentration of 1.5%–2% following rapid induction. The chest area was shaved, and the trunk was positioned at a 30° left tilt. An ultrasound coupling agent was applied to the depilated region, and the ultrasound probe was placed on the left side of the sternum to measure the left ventricular end-diastolic internal diameter (LVEDD) and the left ventricular end-systolic internal diameter (LVESD) using both 2-dimensional and M-mode imaging. The left ventricular ejection fraction (LVEF) was calculated according to the Teichholz formula. Data collection and analysis were conducted using a single-blind method, ensuring that the analysts were unaware of the ultrasound data collection.

HE staining was employed to observe the morphology and structure of myocardial tissues and to initially evaluate the structural changes in the myocardium. The hearts of rats were excised, rinsed with PBS buffer, and infarcted tissues below the ligation line were collected. These tissues were then fixed in a 4% paraformaldehyde solution, routinely embedded in paraffin, sectioned to a thickness of 6 µm, de-waxed with xylene, dehydrated using a gradient of ethanol, and subsequently stained with hematoxylin-eosin. Finally, the slices were sealed with neutral gum and examined under a light microscope to observe the morphological changes in myocardial infarction tissues across the different groups.

ELISA was performed to measure the levels of IL-6, TNF-α, IL-10, and TGF-β in myocardial tissue. After 96 h of modeling and subsequent anesthesia, the heart was excised. The heart tissue from the infarcted area, located below the ligation line, was homogenized. Following centrifugation at 12,000 rpm for 15 min, the supernatant was collected, and the absorbance was measured at 450 nm to quantify the contents of IL-6, TNF-α, IL-10, and TGF-β, as per the instructions provided with the ELISA kits.

The Western blot method was employed to assess the expression levels of PPARγ and NF-κb proteins in myocardial tissues from the infarcted area. At 96 h post-myocardial ischemia/reperfusion injury (MIRI), three rats from each group were randomly selected following isoflurane anesthesia. The hearts were excised, and myocardial tissues from the anterior wall of the left ventricle, located beneath the ligature line, were carefully removed and rinsed with PBS buffer solution. These tissues were then quick-frozen in liquid nitrogen and subsequently milled. Protein extraction was performed using RIPA lysate, and protein concentrations were quantified using the BCA method. Equal amounts of extracted proteins were subjected to SDS-polyacrylamide gel electrophoresis, followed by electrotransfer onto a PVDF membrane. The membrane was blocked with a 5% skimmed milk sealing solution for 2 h, then washed 2–3 times with 1 × TBST. Primary antibodies against PPARγ (1:1,000) and NF-κb (1:1,000) were applied, and the membrane was incubated overnight at 4 °C. Post-incubation, the membrane was washed three times for 10 min each with TBST. Following this, a secondary antibody was added, and the membrane was incubated at room temperature on a shaker for 2 h, followed by another three washes with TBST for 10 min each. Chemiluminescence reagents were utilized to develop the image, with β-actin serving as the internal reference. The gray values of the bands were quantified, and the relative expression levels were determined by analyzing the ratio of the gray values of the target proteins to that of the internal reference protein using ImageJ software.

The expression of Arg-1 and iNOS in myocardial tissue was detected using immunohistochemistry (IHC). Ninety-six hours after myocardial ischemia-reperfusion injury (MIRI), the hearts of three rats from each group were excised following isoflurane anesthesia. The tissue from the anterior wall of the left ventricle, located under the ligature line, was removed, fixed in 4% paraformaldehyde, embedded in paraffin wax, and sliced to a thickness of 5 µm after 24 h. The slices were then unfolded by heating in a water bath at 50 °C and adhered to glass slides. Deparaffinization was performed using xylene I and II, followed by immersion in anhydrous, 95%, 85%, and 70% ethanol, with subsequent washing twice in distilled water and PBS buffer. Following heat-induced antigen retrieval in citrate buffer for 20 min, the sections were rinsed with PBS and treated with 3% hydrogen peroxide for 15 min to eliminate endogenous peroxidase activity, followed by three rinses with PBS. Normal goat serum sealing solution was applied dropwise to the sections for 20 min at room temperature. Subsequently, primary antibodies for iNOS and Arg-1 were added dropwise and incubated overnight at 4 °C, after which the sections were washed with PBS three times for 5 min each. A secondary antibody was then applied dropwise and incubated at 37 °C for 30 min, followed by three additional washes with PBS for 5 min each. Color development was achieved using DAB with H2O for 6 min, and the sections were thoroughly washed with water. Hematoxylin was used for staining for 1 min, followed by thorough washing, differentiation with 1% hydrochloric acid ethanol, and returning to blue with 1% ammonia. The sections were sequentially dehydrated in 70%, 85%, and 100% ethanol, cleared with xylene for 5 min, and finally sealed with neutral resin for microscopic observation. Cells exhibiting positive expression appeared brown, and the percentage of positively expressed cells was quantified.

Macrophage polarization was assessed using flow cytometry. Following the execution of each group of rats, the heart was excised via an open-chest procedure. The infarcted myocardial tissue located beneath the ligation line was extracted, and collagen was digested at room temperature for 1 h after clipping (type I collagenase was added to RPMI-1640 medium at a concentration of 1.6 g/L). After grinding, the collagen was filtered through a 300-mesh filter, centrifuged (1,500 r/min, 4 °C, for 5 min), and the supernatant was discarded. The pellet was resuspended in 1 ml of PBS and subjected to further centrifugation, after which the supernatant was again discarded. Subsequently, 500 µl of erythrocyte lysate was added, and the erythrocytes were lysed for 5 min at 4 °C under light protection. Following this, the supernatant was discarded by centrifugation, and the cells were resuspended with 500 µl of PBS. The cells were then aliquoted into tubes, and the Fc receptors on the surface of the macrophages were blocked using CD16/32 for 5 min. After this step, surface antibody staining was conducted for 40 min. M1-type macrophages were labeled with F4/80+CD11bCD86, while M2-type macrophages were labeled with F4/80+CD11bCD163. Following the addition of 500 µl of PBS to wash away the surface antibodies, the cells were centrifuged, and the supernatant was discarded. The cells were then resuspended in 200 µl of PBS and analyzed using FlowJo software to determine the percentage of cardiac macrophages and the distribution of macrophages of different phenotypes.

### 2.6 Statistical analysis

Statistical analyses were conducted using SPSS version 26.0. Measurements that conformed to a normal distribution were expressed as mean ± standard deviation (*x* ±* s*). One-way ANOVA was employed for comparisons between groups with uniform variance, while the Kruskal–Wallis test was utilized for groups with unequal variance. A difference was considered statistically significant at *P* < 0.05. Additionally, GraphPad version 8 software was used to summarize the data and create statistical graphs.

## 3 Results

### 3.1 Comparison of echocardiographic EF in rats of each group

**Figure 2:**
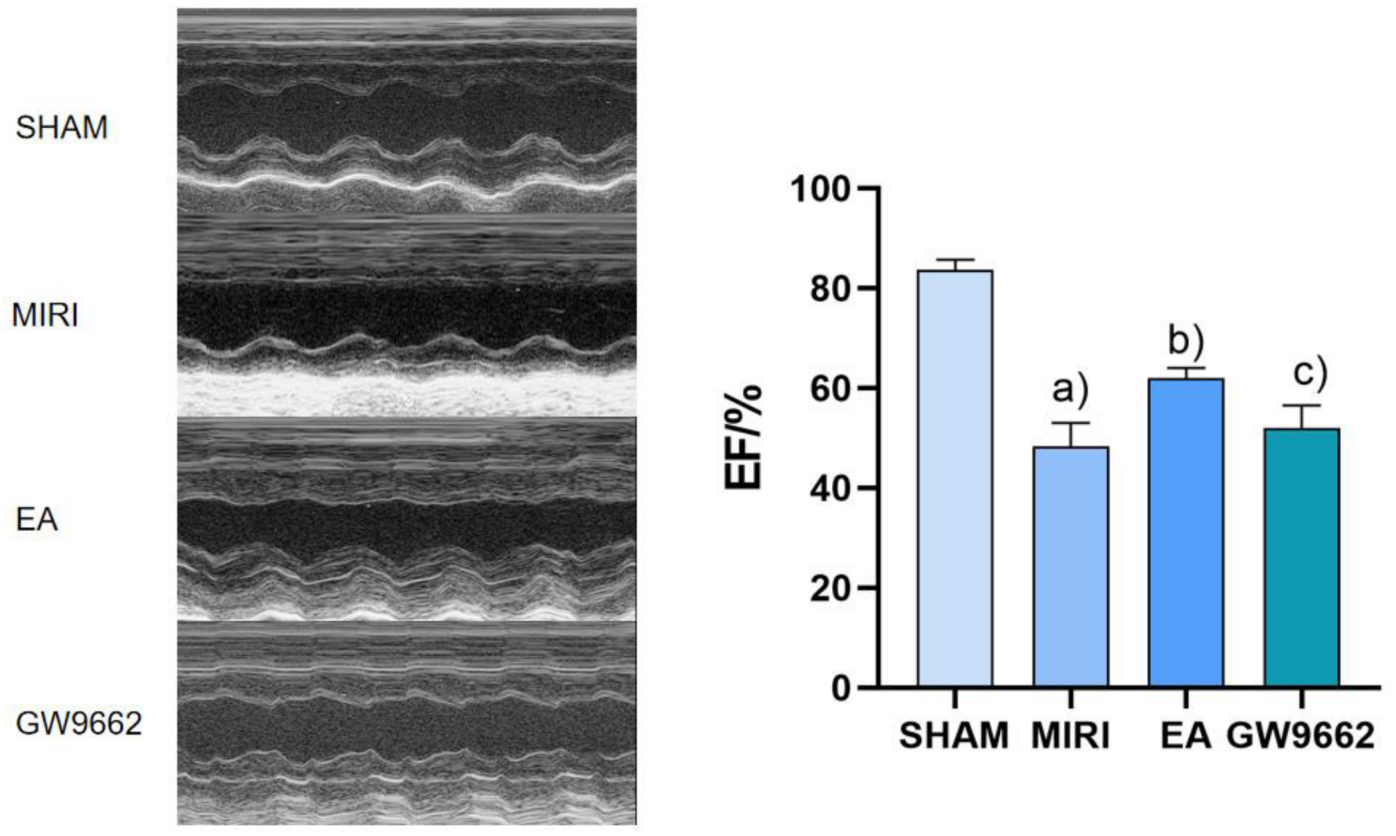
Comparison of left ventricular EF after intervention in groups of rats (*x* ±* s*, 12 rats/group). **(a)** In contrast, the left ventricular ejection fraction (EF) of rats in the model group was significantly lower than that of the sham operation group (*P* < 0.01). **(b)** And the EF of rats in the electroacupuncture group was significantly higher than that of the model group (*P* < 0.01). **(c)** Additionally, the EF of rats in the inhibitor group was lower than that of the electroacupuncture group (*P* < 0.05).

In contrast, the left ventricular ejection fraction (EF) of rats in the model group was significantly lower than that of the sham operation group (*P* < 0.01). And the EF of rats in the electroacupuncture group was significantly higher than that of the model group (*P* < 0.01). Additionally, the EF of rats in the inhibitor group was lower than that of the electroacupuncture group (*P* < 0.05). Refer to Figure 2.

**Figure 3:**
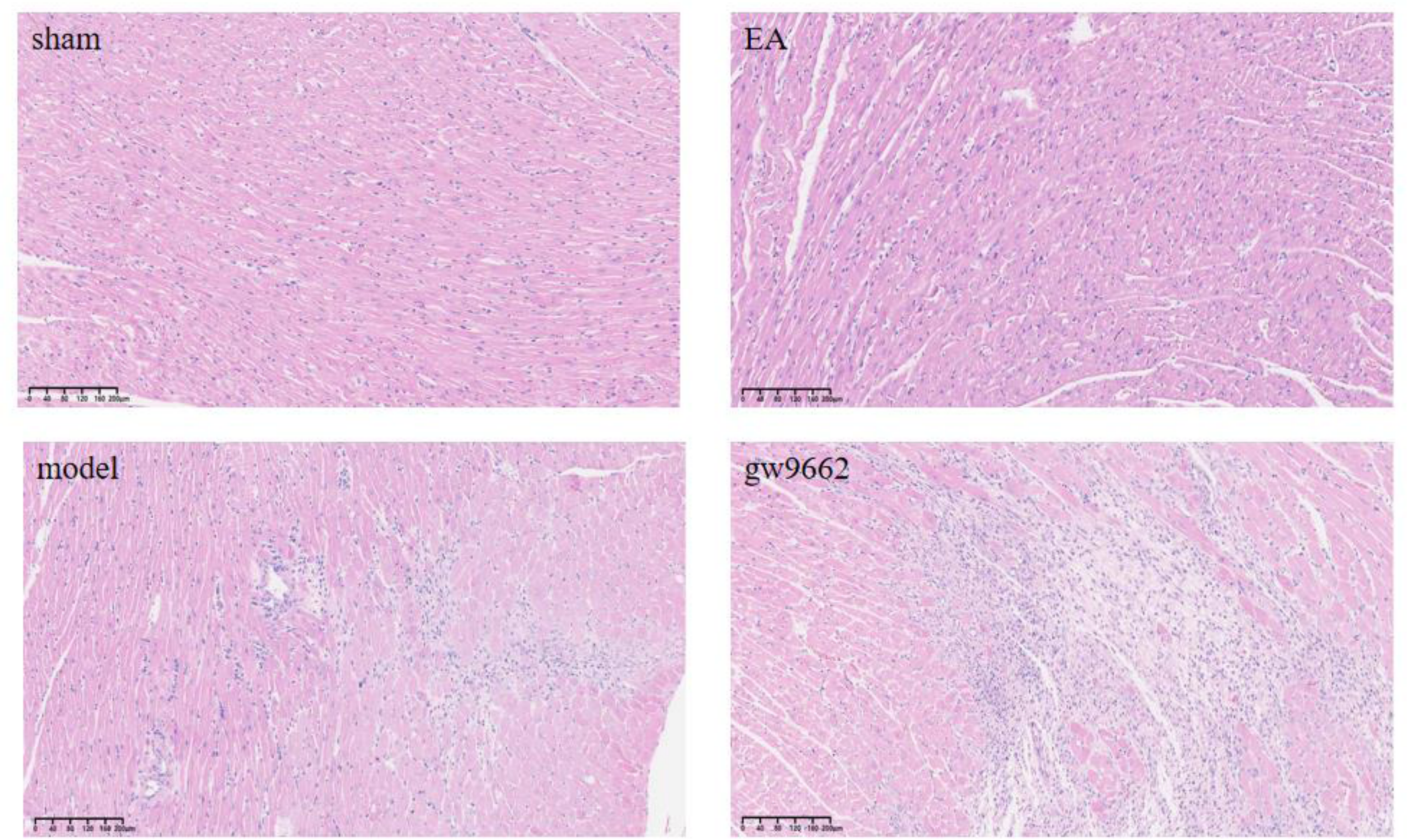
Morphological changes in myocardial infarction tissue after intervention in rats of all groups (HE staining). No inflammatory cell infiltration in sham group; significant inflammatory cell infiltration in MIRI group; improved inflammatory cell infiltration in EA group; significant inflammatory cell infiltration in GW9662 group.

### 3.2 Comparison of histomorphologic changes of myocardial infarction area in rats in various groups

The myocardial fibers of rats in the sham-operated group exhibited a clear and orderly arrangement, with only slight swelling and minimal inflammatory cell infiltration. In contrast, the myocardial fibers of rats in the model and inhibitor groups were disorganized and showed a significant increase in inflammatory cell infiltration. Notably, compared to the model group, the myocardial fiber damage in the electroacupuncture group was reduced, along with a decrease in inflammatory cell infiltration, as illustrated in Figure 3.

### 3.3 Detection of Il-6, TNF-α, IL-10, and TGF-β in myocardial tissue of rats in each group

ELISA results indicated that normal levels of IL-6, TNF-α, IL-10, and TGF-β.

**Figure 4:**
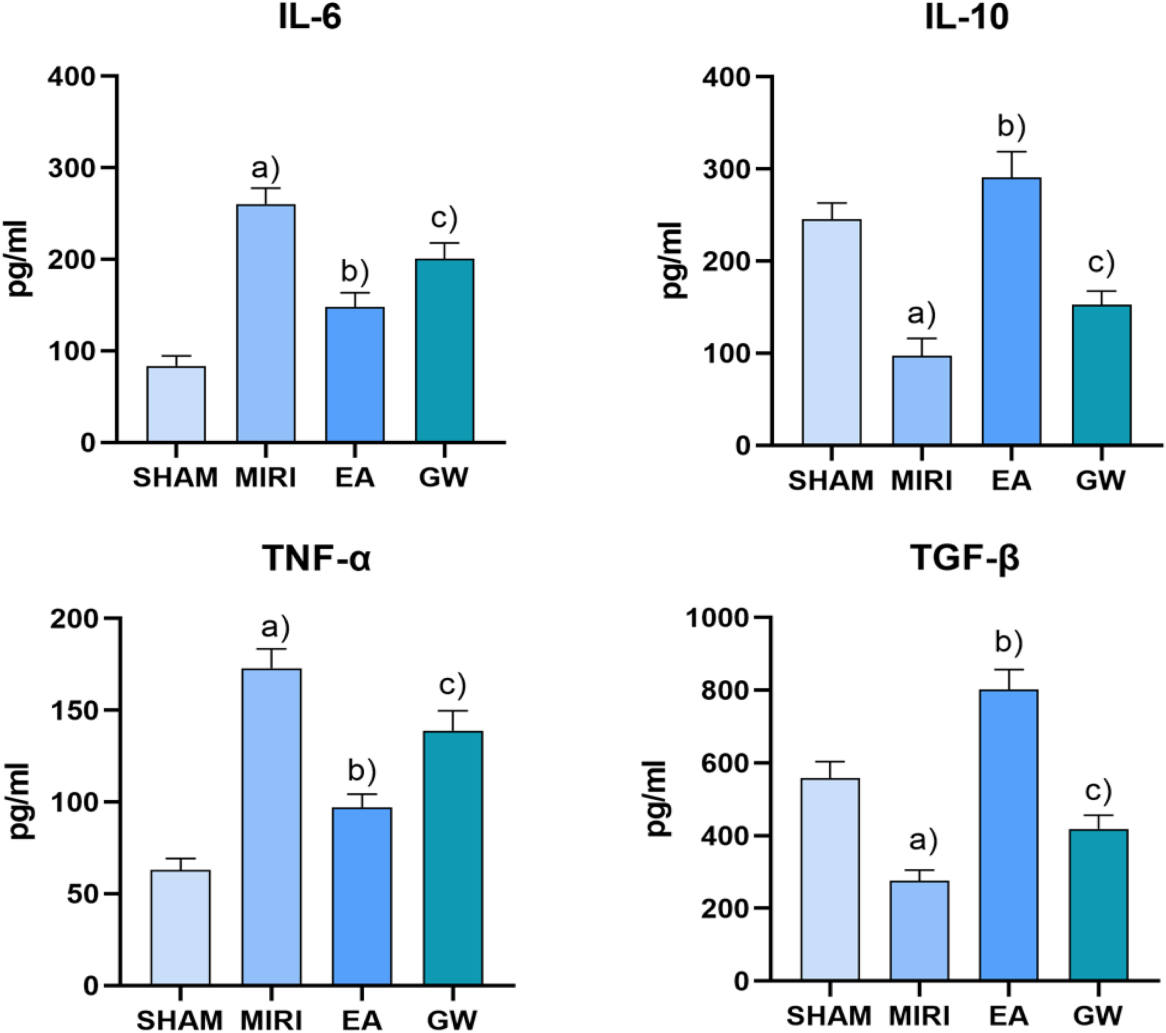
Comparison of serum levels of IL-6, TNF-α, IL-10, and TGF-β in rats in each group after intervention (*x* ±* s*, 3 rats/group). **(a)** In contrast, myocardial tissue IL-6 and TNF-α levels significantly increased and IL-10 and TGF-β levels decreased in the MIRI rats (*P* < 0.01). **(b)** When compared to the MIRI group, myocardial tissue IL-10 and TGF-β levels increased while IL-6 and TNF-α levels decreased in the EA group (*P* < 0.01). **(c)** Furthermore, relative to the EA group, the GW9662 group exhibited increased myocardial tissue IL-6 and TNF-α levels and decreased IL-10 levels and TGF-β (*P* < 0.01).

The results show that, there were present in the myocardial tissue of rats from the sham-operated group. In contrast, myocardial tissue IL-6 and TNF-α levels significantly increased and IL-10 and TGF-β levels decreased in the MIRI rats (*P* < 0.01). When compared to the MIRI group, myocardial tissue IL-10 and TGF-β levels increased while IL-6 and TNF-α levels decreased in the EA group (*P* < 0.01). Furthermore, relative to the EA group, the GW9662 group exhibited increased myocardial tissue IL-6 and TNF-α levels and decreased IL-10 levels and TGF-β (*P* < 0.01). Refer to Figure 4.

### 3.4 Comparison of PPARγ and p-p65 protein expression in myocardial infarction tissues of rats in various groups

**Figure 5:**
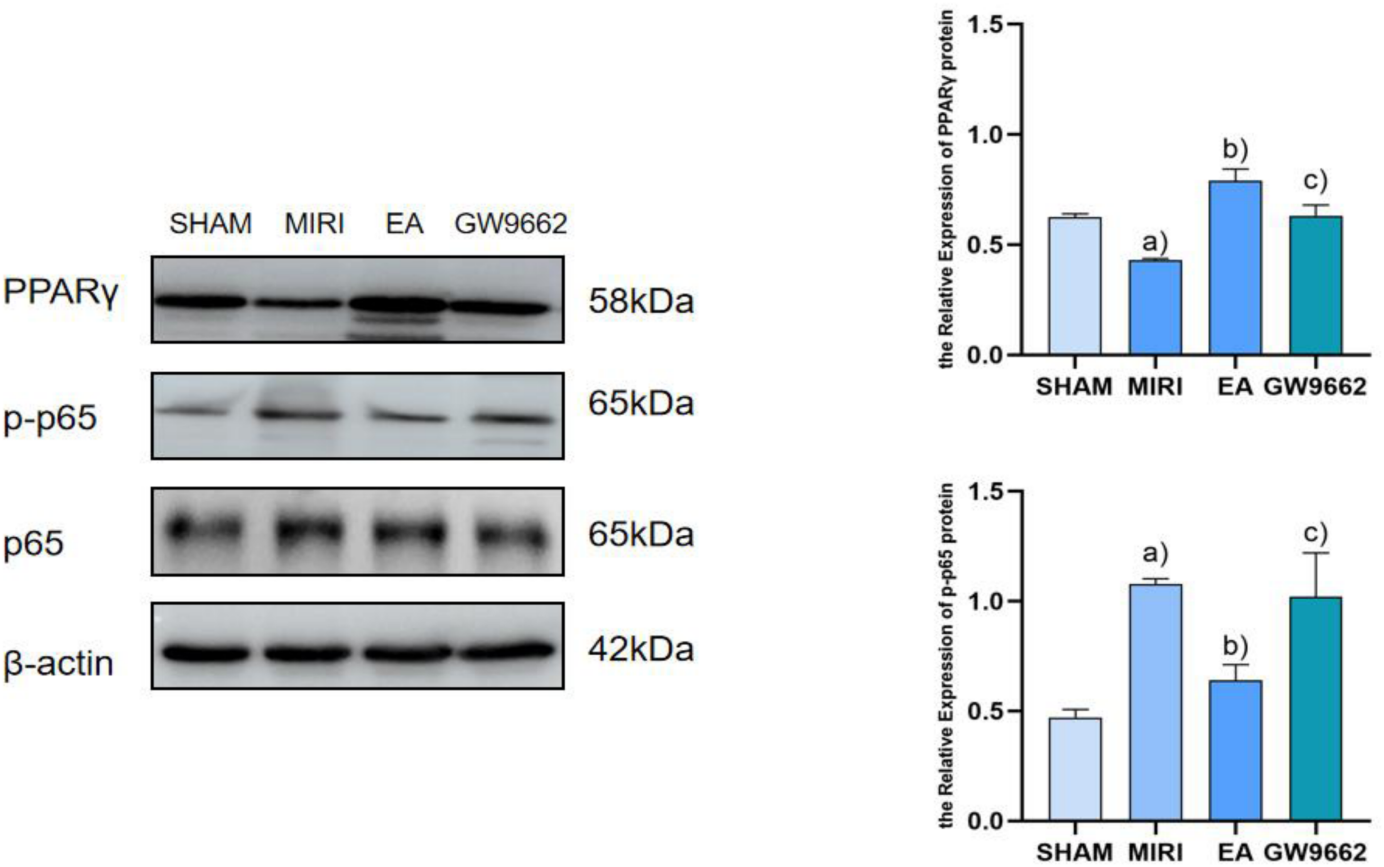
Comparison of PPARγ and p-p65 protein expression in myocardial infarction tissues after intervention in each group of rats (*x* ±* s*, 3 rats/group). **(a)** In comparison to the sham-operated group, p-p65 protein expression in the infarcted tissues of rats in the model group was significantly elevated (*P* < 0.01), while PPARγ protein expression was notably decreased (*P* < 0.01). **(b)** When compared to the model group, p-p65 protein expression in the myocardial infarcted tissues of rats in the electroacupuncture group was reduced (*P* < 0.01), and PPARγ protein expression was increased (*P* < 0.01). **(c)** Furthermore, relative to the electroacupuncture group, the inhibitor group exhibited elevated p-p65 protein expression (*P* < 0.01) and decreased PPARγ protein expression (*P* < 0.01).

In comparison to the sham-operated group, p-p65 protein expression in the infarcted tissues of rats in the model group was significantly elevated (*P* < 0.01), while PPARγ protein expression was notably decreased (*P* < 0.01). When compared to the model group, p-p65 protein expression in the myocardial infarcted tissues of rats in the electroacupuncture group was reduced (*P* < 0.01), and PPARγ protein expression was increased (*P* < 0.01). Furthermore, relative to the electroacupuncture group, the inhibitor group exhibited elevated p-p65 protein expression (*P* < 0.01) and decreased PPARγ protein expression (*P* < 0.01). Refer to Figure 5.

### 3.5 Comparison of Arg-1 and iNOS expression in myocardial tissues of rats in each group

**Figure 6:**
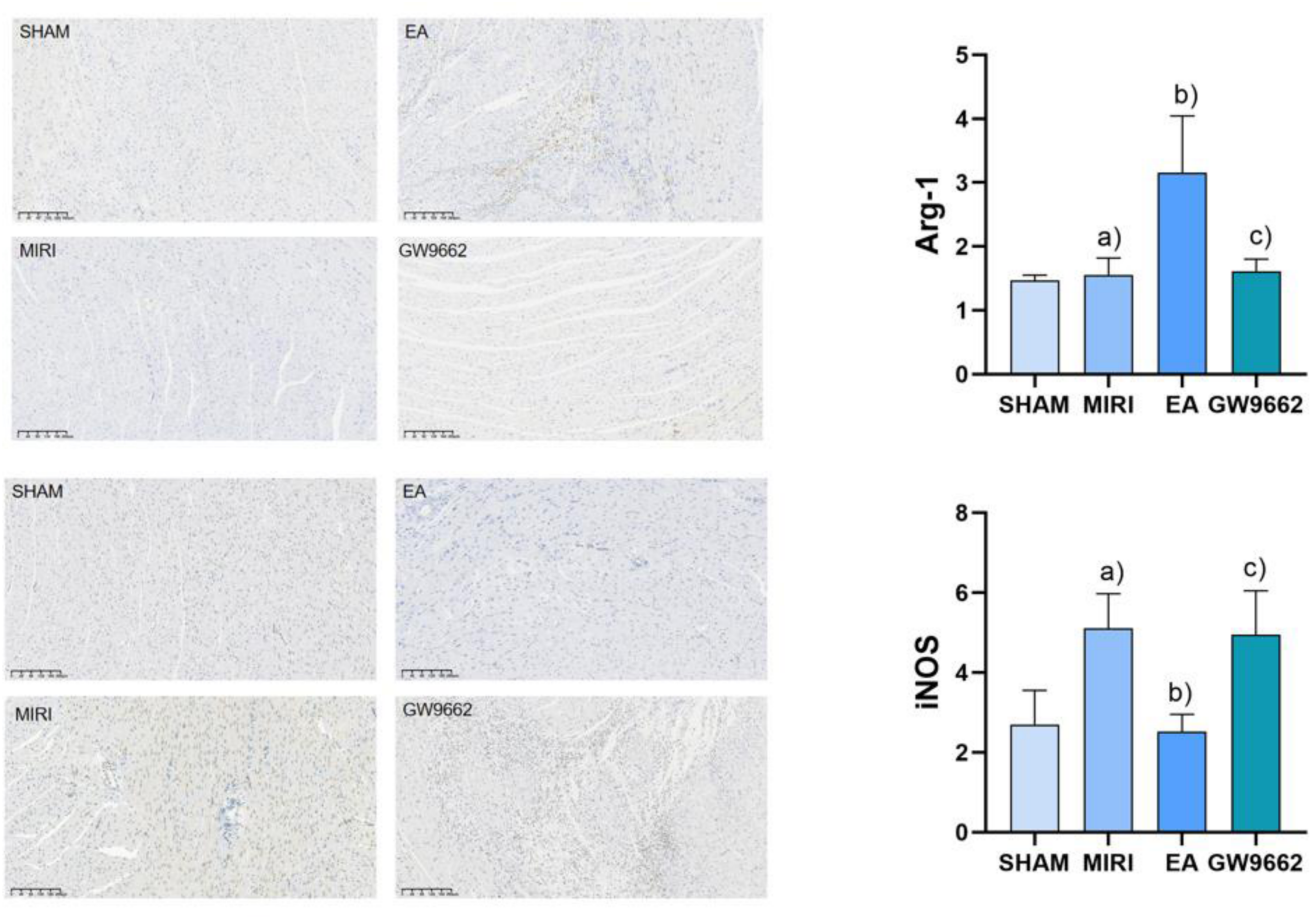
Comparison of Arg-1 and iNOS expression in myocardial tissues of rats in each group. **(a)** In comparison to the sham-operated group, the expression of iNOS in the infarcted tissues of rats in the model group was significantly increased (*P* < 0.05). **(b)** Additionally, relative to the model group, Arg-1 expression in the infarcted tissues of rats in the electroacupuncture group was significantly elevated (*P* < 0.05), while iNOS expression was significantly decreased (*P* < 0.05). **(c)** Furthermore, when comparing the electroacupuncture group to the GW9662 group, Arg-1 expression in the infarcted tissues of rats was significantly reduced (*P* < 0.05), whereas iNOS expression was significantly increased (*P* < 0.05). Refer to Figure 6.

In comparison to the sham-operated group, the expression of iNOS in the infarcted tissues of rats in the model group was significantly increased (*P* < 0.05). Additionally, relative to the model group, Arg-1 expression in the infarcted tissues of rats in the electroacupuncture group was significantly elevated (*P* < 0.05), while iNOS expression was significantly decreased (*P* < 0.05). Furthermore, when comparing the electroacupuncture group to the GW9662 group, Arg-1 expression in the infarcted tissues of rats was significantly reduced (*P* < 0.05), whereas iNOS expression was significantly increased (*P* < 0.05). Refer to Figure 6.

### 3.6 Effect of electroacupuncture on the polarization of macrophages detected by flow cytometry

**Figure 7:**
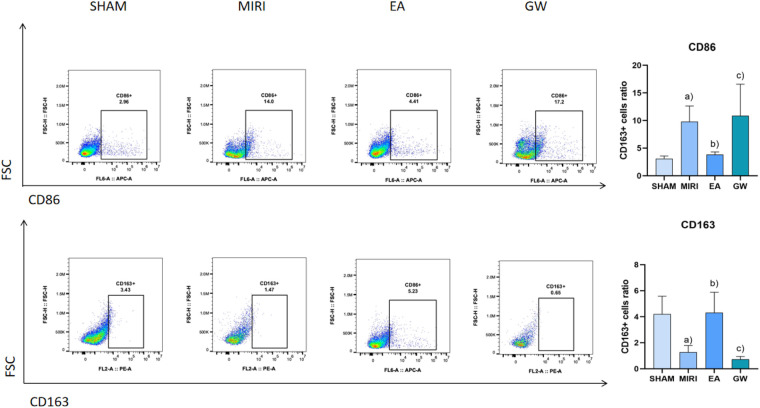
Comparison of macrophage polarization in myocardial tissues of rats from various groups (*x* ±* s*, 3 rats/group). **(a)** Compared with the sham group, macrophage marker CD86 significantly increased (*P* < 0.05) and CD163 significantly decreased (*P* < 0.05) in the model group; macrophage marker CD86 significantly decreased (*P* < 0.05) and CD163 significantly increased (*P* < 0.05) in the electroacupuncture group; **(b)** compared with the electroacupuncture group, macrophage marker CD86 significantly increased (*P* < 0.05) and CD163 significantly decreased (*P* < 0.05); **(c)** compared with the electroacupuncture group, macrophage marker CD86 in the inhibitor group significantly increased (*P* < 0.05).

Compared with the sham group, macrophage marker CD86 significantly increased (*P* < 0.05) and CD163 significantly decreased (*P* < 0.05) in the model group; macrophage marker CD86 significantly decreased (*P* < 0.05) and CD163 significantly increased (*P* < 0.05) in the electroacupuncture group; compared with the electroacupuncture group, macrophage marker CD86 significantly increased (*P* < 0.05) and CD163 significantly decreased (*P* < 0.05); compared with the electroacupuncture group, macrophage marker CD86 in the inhibitor group significantly increased (*P* < 0.05). Refer to Figure 7.

## 4 Discussion

According to the *China Health Statistics Yearbook 2022*, the coronary heart disease mortality rate among Chinese urban residents was 165.37 per 100,000 in urban areas and 188.58 per 100,000 in rural areas in 2021. This mortality rate has continued its upward trend since 2012, with a notable increase in rural areas, which surpassed urban levels by 2016 (21). Consequently, coronary heart disease remains a critical public health issue that requires urgent attention. Reperfusion therapy is a crucial intervention for treating myocardial ischemia, as it restores blood flow to the ischemic regions of the heart following interventional or thrombolytic procedures. However, in some patients, this treatment can lead to more severe injuries than those caused by ischemia itself, including cardiac arrhythmias, an increased area of infarction, and diastolic dysfunction (22). These complications result in myocardial ischemia-reperfusion injury (MIRI). Research suggests that the onset of MIRI may be linked to a significant increase in oxygen free radicals, an inflammatory response, calcium ion overload, apoptosis, and alterations in mitochondrial permeability (23).

Macrophages play a crucial role in the formation of myocardial ischemia-reperfusion injury (MIRI) and its associated inflammatory processes. Under varying stimulation conditions, macrophages can differentiate primarily into two types: M1, which promotes inflammation, and M2, which inhibits inflammation (24, 25). M1-type macrophages secrete pro-inflammatory substances such as IL-6, participate in antigen presentation, and express the marker iNOS, which is primarily involved in inflammatory responses and immune defense. Conversely, M2-type macrophages secrete anti-inflammatory substances like IL-10 and express the marker Arg-1, which contributes to anti-inflammatory responses, tissue repair, and the promotion of pro-angiogenic factors and cytokines (26). The balance between M1 and M2 macrophages largely determines the trajectory of MIRI-related inflammation, neovascularization stability, and healing outcomes. Therefore, strategies aimed at inhibiting macrophage polarization toward the M1 phenotype and promoting differentiation toward the M2 phenotype are critical for controlling inflammation and the progression of MIRI. The findings of this study indicate that electroacupuncture treatment significantly reduced levels of the M1-type pro-inflammatory factor IL-6, TNF-α and the M1-type macrophage marker iNOS, CD86, while significantly increasing levels of the M2-type anti-inflammatory factor IL-10, TGF-β and the M2-type macrophage marker Arg-1, CD163 compared to the model group. Electroacupuncture may enhance MIRI outcomes by competitively upregulating Arg-1, CD163 levels, inhibiting iNOS, CD86 substrate binding, and increasing neovascularization stability. These results confirm that electroacupuncture at the Neiguan point effectively reduces the expression of M1-type macrophages, increases the expression of M2-type macrophages, improves the macrophage polarization ratio, inhibits the intravascular inflammatory response, and subsequently slows the progression of MIRI, thereby achieving therapeutic benefits.

PPARγ is a ligand-dependent activator of nuclear hormone receptors that play crucial roles in cardiovascular protection, the inhibition of inflammatory cell and chemokine release, and the intervention in inflammation formation (27, 28). Several studies have demonstrated (29, 30) that the activation of PPARγ offers protective effects against myocardial ischemia-reperfusion injury (MIRI). Specifically, it has been shown (31) that during MIRI, the activation of PPARγ can inhibit NF-κB activity, thereby exerting anti-apoptotic and anti-inflammatory effects. NF-κB is a vital transcription factor that regulates the expression of inflammatory factors; under normal conditions, NF-κB remains inactive in the cytoplasm. Upon stimulation, NF-κB is released and translocates into the nucleus, where it binds to genes containing κB sites and induces transcriptional effects (32). PPARγ negatively regulates immune and inflammatory responses by binding to NF-κB, preventing its nuclear translocation, and thereby antagonizing the NF-κB signaling pathway. This action attenuates the inflammatory response and exerts a protective effect on the organism. The results of this study indicate that, compared to the blank group, PPARγ expression was significantly lower and NF-κB expression was significantly higher in the model group. Conversely, in the electroacupuncture group, PPARγ expression was significantly higher while NF-κB expression was significantly lower, suggesting that electroacupuncture may inhibit inflammatory responses through the PPARγ/NF-κB signaling pathway. The PPARγ and NF-κB signaling pathways play crucial roles in macrophage polarization, with their interactions regulating the transition of macrophages from a pro-inflammatory (M1-type) to an anti-inflammatory (M2-type) phenotype. PPARγ exerts an anti-inflammatory effect by inhibiting the NF-κB signaling pathway, which promotes M2-type polarization, whereas the activation of NF-κB facilitates M1-type polarization (10). Following electroacupuncture intervention, the PPARγ/NF-κB signaling pathway may have been activated, resulting in an increase in aortic PPARγ protein expression and a decrease in NF-κB protein levels. This change is accompanied by a significant reduction in the levels of downstream factors, such as iNOS, CD86, TNF-α and IL-6, alongside a notable increase in Arg-1, TGF-β, CD163 and IL-10 levels. These findings suggest that the PPARγ/NF-κB signaling pathway facilitates a shift towards M2 macrophage polarization by inhibiting M1 macrophage polarization, thereby contributing to the amelioration of MIRI.

In summary, the observed decrease in M1-type polarization markers coupled with an increase in M2-type polarization markers following electroacupuncture treatment indicates that electroacupuncture may influence myocardial ischemia-reperfusion injury (MIRI) by modulating the M1/M2 polarization dynamics of macrophages. Furthermore, the PPARγ/NF-κB signaling pathway may play a significant role in the activation of macrophages and could contribute to the amelioration of MIRI by promoting the polarization of macrophages towards the M2 phenotype. This experiment establishes a new experimental foundation for acupuncture research by identifying the PPARγ/NF-κB signaling pathway as a key target. However, the conduction of this signaling pathway is inherently complex, suggesting that further investigation into other related signaling pathways and mechanisms is necessary to fully understand the treatment process for MIRI.

## Data Availability

The original contributions presented in the study are included in the article/Supplementary Material, further inquiries can be directed to the corresponding author.

## References

[1] Safi S Sethi NJ Nielsen EE Feinberg J Gluud C Jakobsen JC. Beta-blockers for suspected or diagnosed acute myocardial infarction. Cochrane Database Syst Rev. (2019) 12(12):CD012484. 10.1002/14651858.CD012484.pub2 31845756 PMC6915833

[2] Anderson JL Morrow DA. Acute myocardial infarction. N Engl J Med. (2017) 376(21):2053–64. 10.1056/NEJMra1606915 28538121

[3] Reed GW Rossi JE Cannon CP. Acute myocardial infarction. Lancet. (2017) 389(10065):197–210. 10.1016/S0140-6736(16)30677-8 27502078

[4] Bagai A Dangas GD Stone GW Granger CB. Reperfusion strategies in acute coronary syndromes. Circ Res. (2014) 114(12):1918–28. 10.1161/CIRCRESAHA.114.302744 24902975

[5] Stone GW Selker HP Thiele H Patel MR Udelson JE Ohman EM Relationship between infarct size and outcomes following primary PCI. J Am Coll Cardiol. (2016) 67(14):1674–83. 10.1016/j.jacc.2016.01.069 27056772

[6] Liu F-Y Fan D Yang Z Tang N Guo Z Ma S-Q TLR9 is essential for HMGB1-mediated post-myocardial infarction tissue repair through affecting apoptosis, cardiac healing, and angiogenesis. Cell Death Dis. (2019) 10(7):480. 10.1038/s41419-019-1718-7 31209243 PMC6579765

[7] De Couto G. Macrophages in cardiac repair: environmental cues and therapeutic strategies. Exp Mol Med. (2019) 51(12):1–10. 10.1038/s12276-019-0269-4 31857583 PMC6923399

[8] Yue Y Huang S Wang L Wu Z Liang M Li H M2b macrophages regulate cardiac fibroblast activation and alleviate cardiac fibrosis after reperfusion injury. Circ J. (2020) 84(4):626–35. 10.1253/circj.CJ-19-0959 32161201

[9] Gurevich DB Severn CE Twomey C Greenhough A Cash J Toye AM Live imaging of wound angiogenesis reveals macrophage orchestrated vessel sprouting and regression. EMBO J. (2018) 37(13):e97786. 10.15252/embj.201797786 29866703 PMC6028026

[10] Chen J Xuan Y Chen Y Wu T Chen L Guan H Netrin-1 alleviates subarachnoid haemorrhage-induced brain injury via the PPAR γ/NF-KB signalling pathway. J Cell Mol Med. (2019) 23(3):2256–62. 10.1111/jcmm.14105 30614619 PMC6378208

[11] Song W Zhang Z-Y Wang K Qiu H-R Zhang X-B Shen T. Zhuyu Pills promote polarization of macrophages toward M2 phenotype to prevent atherosclerosis via PPARγ/NF-κB signaling pathway. China J Chinese Materia Medica. (2024) 49(1):243–50. 10.19540/j.cnki.cjcmm.20230823.501 38403357

[12] Mengting F. *Effect of electroacupuncture pretreatment with “symptom-based acupoint matching” on myocardial and sympathetic nerve remodeling in rats with myocardial ischemia-reperfusion model rats* (master's thesis). Hubei University of Chinese Medicine. (2020). 10.27134/d.cnki.ghbzc.2020.000132

[13] Zhang T Yang W Wang Y Yuan J Qian Y Sun Q Electroacupuncture preconditioning attenuates acute myocardial ischemia injury through inhibiting NLRP3 inflammasome activation in mice. Life Sci. (2020) 248:117451. 10.1016/j.lfs.2020.117451 32088213

[14] Gaofeng L Jilong G Yufang J Aiai D Xiaoyun L Yun L Effects of acupoints prescription “Jiangyafang” on myocardial oxidative stress in spontaneously hypertensive rats. Emerg Med Tradit Chin Med. (2020) 29(9):1519–23. 10.3969/j.issn.1004-745X.2020.09.005

[15] Du L. *The effects of pre-treatment with electro-acupuncture and moxibustion on autophagy and apoptosis of cardiac myocytes in rats with myocardial ischemia/reperfusion injury* (master's thesis). Hunan University of Chinese Medicine (2018). CNKI:CDMD:2.1018.190030

[16] Tan C-F Wang C Du L Liu W-W Song J Feng G. Effect of electroacupuncture and moxibustion pretreatment on expression of autophagy related proteins LC 3 and beclin 1 in rats with myocardial ischemiareperfusion injury. J Acupunct Res. (2018) 43(1):1–7. 10.13702/j.1000-0607.170181 29383886

[17] Xiao Y Chen W Zhong Z Ding L Bai H Chen H Electroacupuncture preconditioning attenuates myocardial ischemia-reperfusion injury by inhibiting mitophagy mediated by the mTORC1-ULK1-FUNDC1 pathway. Biomed Pharmacother. (2020) 127:110148. 10.1016/j.biopha.2020.110148 32344255

[18] Chen J Deng L Zhang H Zhao D. Discussion on the therapeutic effects of Neiguan Acupoint in clinical practice. China's Naturopathy. (2018) 26(6):102–3. 10.19621/j.cnki.11-3555/r.2018.0672

[19] Li J. *A comparative study of electroacupuncture on PC6, H7 and LI4 influencing ERK signaling pathways in myocardial hypertrophic rats* (doctor's thesis). Hubei University of Chinese Medicine (2012). 10.7666/d.y2100663

[20] Lu X Li M Zhang Z. Analysis of Neiguan in great compendium of acupuncture and moxibustion. Jilin J Chin Med. (2018) 38(2):236–9. 10.13463/j.cnki.jlzyy.2018.02.034

[21] NHC. 中国卫生健康统计年鉴 (2022). Available online at: http://www.nhc.gov.cn/mohwsbwstjxxzx/tjtjnj/202305/6ef68aac6bd14c1eb9375e01a0faa1fb.shtml (accessed August 28, 2024).

[22] Yang C-F. Clinical manifestations and basic mechanisms of myocardial ischemia/reperfusion injury. Tzu Chi Med J. (2018) 30(4):209. 10.4103/tcmj.tcmj_33_18 30305783 PMC6172894

[23] Liu N-B Wu M Chen C Fujino M Huang J-S Zhu P Novel molecular targets participating in myocardial ischemia-reperfusion injury and cardioprotection. Cardiol Res Pract. (2019) 2019:1–16. 10.1155/2019/6935147 31275641 PMC6558612

[24] Martinez FO Gordon S. The M1 and M2 paradigm of macrophage activation: time for reassessment. F1000Prime Rep. (2014) 6:13. 10.12703/P6-13 24669294 PMC3944738

[25] Zhang F Liu H Jiang G Wang H Wang X Wang H Changes in the proteomic profile during the differential polarization status of the human monocyte-derived macrophage THP-1 cell line. Proteomics. (2015) 15(4):773–86. 10.1002/pmic.201300494 25411139

[26] Jetten N Verbruggen S Gijbels MJ Post MJ De Winther MPJ Donners MMPC. Anti-inflammatory M2, but not pro-inflammatory M1 macrophages promote angiogenesis *in vivo*. Angiogenesis. (2014) 17(1):109–18. 10.1007/s10456-013-9381-6 24013945

[27] Clark RB. The role of PPARs in inflammation and immunity. J Leukocyte Biol. (2002) 71(3):388–400. 10.1189/jlb.71.3.388 11867676

[28] Ota N Soga S Haramizu S Yokoi Y Hase T Murase T. Tea catechins prevent contractile dysfunction in unloaded murine soleus muscle: a pilot study. Nutrition. (2011) 27(9):955–9. 10.1016/j.nut.2010.10.008 21641774

[29] Lin F Xu L Huang M Deng B Zhang W Zeng Z β-sitosterol protects against myocardial ischemia/reperfusion injury via targeting PPAR γ/NF-κB signalling. Evid Based Complement Alternat Med. (2020) 2020:1–9. 10.1155/2020/2679409 32308701 PMC7142345

[30] Khandoudi N Delerive P Berrebi-Bertrand I Buckingham RE Staels B Bril A. Rosiglitazone, a peroxisome proliferator-activated receptor-γ, inhibits the jun NH2-terminal kinase/activating protein 1 pathway and protects the heart from ischemia/reperfusion injury. Diabetes. (2002) 51(5):1507–14. 10.2337/diabetes.51.5.1507 11978649

[31] Xie B Liu X Yang J Cheng J Gu J Xue S. PIAS1 protects against myocardial ischemia-reperfusion injury by stimulating PPARγ SUMOylation. BMC Cell Biol. (2018) 19(1):24. 10.1186/s12860-018-0176-x 30419807 PMC6233564

[32] Yu H. Targeting NF-κB pathway for the therapy of diseases: mechanism and clinical study. Signal Transduct Target Ther. (2020) 5(1):209. 10.1038/s41392-020-00312-6 32958760 PMC7506548

